# Associations Between Feeding Management Practices Across Lactation and Goat Milk Composition in Semi-Intensive Systems: A Structural Equation Modeling Approach

**DOI:** 10.3390/ani16152428

**Published:** 2026-08-06

**Authors:** Vasileios Alexandridis, Eleni Malissiova, Natalia Vasileiou, Dimitrios Kantas

**Affiliations:** Department of Animal Science, University of Thessaly, Gaiopolis, 41500 Larisa, Greece; malissiova@uth.gr (E.M.); dkantas@uth.gr (D.K.)

**Keywords:** dairy goats, milk composition, feeding practices, lactation stage, structural equation modeling

## Abstract

Goat milk composition is an important determinant of the nutritional, technological, and economic value of dairy products and is influenced by feeding management. This study examined the associations between feeding practices implemented during different lactation stages and goat milk quality, expressed as a latent construct comprising fat, protein, lactose, and solids-not-fat, in semi-intensive dairy goat farms in Northern Greece. Data were collected from 242 commercial farms through milk sampling and a structured questionnaire on feeding management, animal characteristics, and farm practices. Associations between feeding practices and milk quality were evaluated using structural equation modeling. The final model showed satisfactory fit and explained 46.6% of the variance in the latent milk-quality construct. Moderate concentrate supplementation (300–500 g/day) during mid and late lactation showed the strongest positive association with milk quality, followed by grass–legume hay, protein concentrate supplementation, dairy mixes containing vitamins and minerals, pasture intake of 3–4 kg/day, and goat balancer use. Annual milk yield was negatively associated with milk quality, whereas goat age showed a positive association. These findings indicate that lactation-stage-specific feeding management emphasizing moderate concentrate supplementation and high-quality forage is associated with improved milk composition and may help optimize feeding strategies in semi-intensive dairy goat systems.

## Simple Summary

The quality of goat milk is important because it influences the nutritional value and processing characteristics of cheese, yogurt and other dairy products. This study investigated the relationships of feeding management practices applied at different lactation stages with goat milk quality in 242 semi-intensive dairy goat farms in Northern Greece. Milk quality was evaluated as a combination of protein, fat, lactose, and solids-not-fat using structural equation modeling. The results showed that moderate concentrate supplementation during mid and late lactation, protein concentrate supplementation during early and late lactation, grass–legume hay, dairy mixes containing vitamins and minerals, and moderate pasture intake were all positively associated with milk quality. In contrast, higher annual milk yield was associated with lower concentrations of milk solids, whereas older goats tended to produce milk of slightly higher quality. These results suggest that adjustment of feeding management to the nutritional needs of different lactation stages is correlated with better milk composition under commercial farm conditions. The results provide practical information that could be useful to farmers to optimize feeding strategies and to improve the technological and nutritional quality of goat milk.

## 1. Introduction

Milk quality is a decisive factor in determining both the nutritional value and the economic importance of dairy products. In goat farming, milk composition directly influences consumer acceptance and the processing efficiency of derivatives such as cheese and yogurt [1,2]. The nutritional components—fat, protein, lactose, and solid non-fat (SNF)—are regarded as key measurable indicators [3,4]. These features are essential for human nutrition and affect the technological efficiency of milk in dairy processing [4], as well as its economic value in local and global markets [5].

Several factors influence milk quality, including genetics, management practices, and environmental conditions [3,6]. Nutritional strategies during critical physiological stages are especially important, as they directly shape milk composition [7] and establish the foundation for future milk yield [8]. The lactation stage is a significant determinant that affects feeding outcomes. Studies indicate that nutritional utilization and milk component synthesis vary across different stages of lactation [9,10,11]. These dynamics highlight the necessity for stage-specific feeding regimens to enhance production and milk quality outcomes.

Numerous studies have focused on specific nutrients or supplementation methods (such as protein, lipids, fiber, energy density, plant oils, seeds, microalgae, etc.) and evaluated direct milk attributes (including fat, protein, lactose, and fatty acid profiles). These investigations generally utilize conventional ANOVA/GLM or regression analyses to investigate the direct relationships between feeding parameters and milk components (e.g., [12,13]). While these methodologies have produced significant findings, they do not consider the interconnections between nutrition, animal physiology, lactation stage, and management practices [14,15,16].

Moreover, the systemic impact of feeding practices-considering seasonal variations, environmental factors, and the complex interactions between physiological and managerial variables-remains to be fully understood. Recent studies indicate that although various dietary treatments, such as bypass fats or targeted supplementation, may influence specific compositional qualities, their outcomes often show inconsistency across different contexts [3,17]. The intricacy of diet-environment interactions and the diversity of outcomes underscore the necessity for more comprehensive methodologies capable of elucidating the cumulative and indirect effects of feeding practices on overall milk quality [18,19].

To address these methodological constraints—including the inability of traditional models to capture the systemic, context-sensitive effects of feeding practices—a recent study has emphasized the value of multivariate and latent-variable approaches. Structural equation modeling (SEM) is particularly advantageous due to its capacity to estimate both direct and indirect relationships between latent and observable variables simultaneously. Structural Equation Modeling (SEM) has been utilized in dairy research to analyze complex trait sets, which include milk health indicators, coagulation characteristics, and farm management techniques; this approach reveals relationships that more simplistic models might overlook [20,21]. Yalçin [22] has shown that partial least squares SEM is effective in uncovering latent characteristics, including sensory attributes, microbiological quality, and freshness. The results of these studies demonstrate that advanced models can elucidate the intricate attributes of milk quality.

This issue is particularly relevant in Central Macedonia, Greece, where semi-intensive goat production systems predominate. Farmers commonly rely on a mixture of natural pasture, stored forages, and supplemental concentrates. Diets are frequently adjusted with protein-rich feeds to meet changing demands across lactation stages, making the region a pertinent case for evaluating how feeding strategies influence milk composition and quality outcomes. Understanding how to enhance milk quality through tailored feeding strategies has implications that extend beyond the profitability of the farm. It is in direct alignment with the strategic objectives of the European Union and the Mediterranean region to promote sustainable agrarian development [23,24]. These objectives necessitate livestock production models that minimize dependence on external inputs and optimize resource utilization on the farm [25].

The current study aims to investigate the impact of feeding practices during the early, mid-, and late lactation periods on the quality of goat milk, which is defined by fat, protein, lactose, and solid non-fat (SNF) content. Structural equation modeling (SEM) analyzes the complex interactions among feeding practices, physiological factors (including age and yield), and management practices, and their impact on milk quality. The findings offer practical insights into stage-specific feeding regimens that improve yield and compositional quality.

## 2. Conceptual Framework

This study uses a systems theory approach to describe milk quality as a multidimensional construct defined by composition indicators including fat, protein, lactose, and solid non-fat (SNF). It emphasizes that goat milk quality is the result of complex interactions between feeding strategies, animal physiology, and management decisions, rather than the effect of individual nutrients alone. Using structural equation modeling (SEM) is both a statistical choice and a theoretical necessity because it simultaneously estimates the relationships among feeding-management practices, biological covariates, and the latent construct of milk quality within a single analytical framework. This approach is particularly appropriate for semi-intensive production systems, where feeding decisions, physiological characteristics, and management practices are closely interconnected. The current section delineates the specific variables utilized in the research, categorized into three domains: (i) feeding practices (nutritional inputs pertinent to lactation), (ii) decision-making regarding feeding and milking and hygiene management, and (iii) physiological variables.

### 2.1. Biological Basis of the Milk Quality Construct

Milk quality is a multidimensional concept encompassing the nutritional, physicochemical, and technological characteristics of milk that collectively determine its value for human consumption and dairy processing. Because milk quality cannot be measured directly by a single variable, it is commonly evaluated through a set of compositional indicators representing its principal chemical constituents. In dairy science, milk fat, protein, lactose, and solids-not-fat (SNF) are universally recognized as the primary indicators of milk composition and quality because they reflect nutritional value, technological performance, and commercial grading standards. These constituents are routinely used in quality-control programs [26,27,28], dairy breeding [29,30], nutritional studies-especially in animal milk composition research and in human milk macronutrient analyses [31,32,33]-and assessments of processing suitability, particularly for cheese manufacture [1,34,35]. Consequently, the present study conceptualized milk quality as a latent construct represented by these four observable compositional indicators, which were selected a priori based on established biological knowledge rather than on statistical considerations alone.

Milk fat is one of the most economically important constituents of goat milk because it contributes substantially to its energy content, sensory characteristics, texture, flavor, and cheese yield [5,34,36]. Fat composition is also highly responsive to nutritional management, particularly dietary forage-to-concentrate ratio, energy supplementation, and grazing practices, making it a sensitive indicator of feeding-related changes in milk composition [37,38]. Numerous studies have demonstrated that dietary manipulation can substantially modify milk fat concentration and fatty acid profile, thereby influencing both the nutritional and technological quality of goat milk [19,37,39]. Because fat responds dynamically to nutritional management while contributing directly to product quality and consumer acceptance, it represents an essential component of any comprehensive evaluation of milk quality.

Milk protein constitutes the second major nutritional component of goat milk and is particularly important for cheese production because casein proteins largely determine coagulation properties, cheese yield, and processing efficiency [36,40]. Protein concentration is influenced by dietary protein supply [7], energy balance [41], physiological status [42], and lactation stage [43,44], making it a valuable indicator of both nutritional adequacy and productive performance. From a nutritional perspective, goat milk proteins provide highly digestible amino acids [45] and contribute substantially to the biological value of milk [1]. Their importance for both nutritional quality and dairy processing has resulted in protein concentration being consistently included among the principal indicators used to characterize goat milk quality across breeds, production systems, and environmental conditions [7].

Lactose, the principal carbohydrate of milk, plays a fundamental physiological role in regulating mammary gland osmotic pressure and milk secretion [46,47]. Unlike fat and protein, lactose concentration is under tighter homeostatic regulation because it is directly associated with milk volume and water transport within the mammary gland [47,48]. Consequently, previous studies have consistently reported considerably lower biological variability for lactose than for fat and protein across breeds, farming systems, and lactation stages. For example, Vacca et al. [35] reported a coefficient of variation in only approximately 6% for lactose compared with substantially higher variability for fat and protein, while Pazzola et al. [34] similarly observed a relatively narrow distribution of lactose concentrations under different production systems. This physiological stability may reduce the statistical covariance of lactose with other compositional traits, thereby resulting in lower factor loadings in latent-variable models. Nevertheless, lactose remains one of the four principal chemical constituents routinely determined in dairy laboratories and constitutes an indispensable component of milk composition [3,35,46,49]. Therefore, despite its expected lower variability, its inclusion in the latent construct is biologically justified and preserves the conceptual completeness of milk quality.

Solids-not-fat (SNF) represent all milk solids excluding fat and comprise proteins, lactose, minerals, vitamins, and other minor constituents. Because SNF integrates several chemically important components into a single measurement, it provides a comprehensive indicator of the nutritional density and compositional integrity of milk [1,50,51]. In commercial dairy production, SNF is widely employed in quality grading, payment systems [50,52], and the evaluation of processing characteristics, particularly in products requiring high dry-matter recovery [53,54]. Variations in SNF reflect the combined influence of nutrition, physiological status, and environmental conditions on overall milk composition and therefore complement information obtained from individual milk constituents [55,56,57]. The inclusion of SNF alongside fat, protein, and lactose enables a more comprehensive assessment of compositional quality than would be achieved by considering individual components alone.

Although these four constituents differ in their physiological regulation and responsiveness to nutritional management, together they provide complementary information regarding the nutritional, physicochemical, and technological characteristics of goat milk. Their combined use is well established in dairy science and has been adopted extensively for evaluating milk composition under different breeds, production systems, feeding strategies, and stages of lactation [3,56,58]. Consequently, the latent variable ‘Quality’ used in the present study was specified on the basis of this established biological framework rather than empirical optimization of the observed data. The subsequent confirmatory factor analysis was therefore undertaken to verify that the hypothesized biologically derived latent construct was adequately represented by these four indicators before estimating the structural relationships specified in the SEM.

### 2.2. Feeding Practices

Milk quality is influenced by feeding practices through two principal biological mechanisms: (a) fermentation in the rumen, where volatile fatty acids aid in de novo milk fat synthesis and propionate provides lactose precursors; and (b) nitrogen and amino acid flow in the rumen, which contributes to casein and total protein synthesis [19]. Within this framework, multiple dimensions of diet are considered, which include forage and grazing systems, concentrate type and proportion, and grazing time and pasture intake.

#### 2.2.1. Forage and Grazing System

Forage and grazing systems constitute the principal nutritional foundation for lactating goats and strongly influence rumen fermentation, nutrient digestibility, and milk composition, particularly fat and protein [59,60]. High-quality pastures, grasslands, and seeded forages generally improve milk fat, protein, and sensory properties, whereas low-quality feeds such as agricultural residues and straw reduce milk solids unless supplemented [61,62]. High-quality alfalfa hay boosts milk fat, protein, and SNF in dairy goats, while low-quality or high-fiber hay can reduce fat yield [63,64]. Adding legumes to forage mixtures improves protein nourishment, nitrogen use, and milk protein and SNF levels. Mid-lactation goats fed mixed hays often show higher protein and SNF [5,65,66]. Assuming a well-balanced diet and good-quality silage, milk fat, protein, and SNF levels tend to remain unchanged. The lactose concentration in milk is mostly unaltered [46]. If silage quality is poor or intake declines, then milk fat yield may decrease, and so SNF may be slightly reduced [12].

#### 2.2.2. Hours of Grazing and Pasture Intake (kg/Day)

Grazing duration (hours) and pasture intake determine total forage dry matter intake (DMI), which directly affects milk yield and indirectly modulates milk composition via nutrient balance and metabolic load [67,68]. These effects are most evident in mid-lactation, when nutrient conversion efficiency and mammary capacity are at their peak [69]. Short grazing durations often do not meet the energy requirements of lactating goats, resulting in lower output and changed fat-to-protein ratios [68]. Moderate grazing duration and intake promote efficient nutrient utilization [67], whereas significant intake variation affects milk composition and may lead to a decline in milk quality because of energy imbalance or over-conditioning [70].

The current study classified pasture intake into six levels (under 2 kg/day to over 6 kg/day) and grazing duration into four levels (under 2 h, 2–4 h, 4–6 h, and over 6 h/day). These levels imply a range of forage availability and consumption, from an insufficient intake for maintenance to a potentially excessive intake that could affect digestibility.

#### 2.2.3. Concentrate Types

Concentrates—such as grains, by-products, protein supplements, and specialized balancers—supply fermentable carbohydrates and digestible protein; their inclusion modifies energy availability, microbial protein synthesis, and the synchrony between rumen nitrogen and energy [71]. Whole grains such as corn, wheat, and oats generally increase milk yield, but excessive use may lower milk fat because they reduce rumen pH [72]. Flours and agro-industrial by-products can improve milk solids when they are well balanced with forage [73]. Soy flour and protein concentrates enhance amino acid supply and usually increase milk protein and solid non-fat, particularly when enough energy is available [74]. Cottonseed cake can increase milk fat and energy intake [75], but its use must be limited due to the risk of gossypol toxicity [76]. Molasses improves feed intake and rumen activity at moderate levels, although high inclusion may reduce milk fat [77]. Vitamin–mineral dairy mixes and goat balancers help correct nutritional deficiencies and stabilize milk composition when used in a targeted manner [78,79]. Pelleted concentrate mixes are common in semi-intensive systems and improve feeding consistency, but very high levels may depress milk fat [14].

#### 2.2.4. Concentrate Proportion and Daily Amount

The proportion of concentrate in the total diet and the daily amount offered are key determinants of nutrient balance, rumen fermentation dynamics, and ultimately milk composition in goats. Concentrate inclusion levels below 20% of total dry matter generally limit energy supply and microbial protein synthesis, often resulting in lower milk yield and protein content [59,64]. Moderate inclusion rates, usually between 30 and 40%, help synchronize rumen-degradable nitrogen and fermentable energy, which leads to better nutrient use and more milk fat and protein synthesis [59,80]. However, when the concentrate proportion exceeds 50% or daily provision surpasses 700 g/goat, the rapid fermentation of starch can decrease rumen pH and fiber digestibility, leading to milk-fat depression and potential metabolic stress [81].

### 2.3. Feeding Management, Milking, and Hygiene Variables

Several studies support the notion that management choices shape feeding regimes and thus production outcomes [82,83,84]. Management rules for farms, such as how concentrates are allocated (e.g., based on yield history), feeding adjustments during lactation (e.g., reducing concentrate as yield declines), and the use of energy supplements in late pregnancy (e.g., feeding adjustment by number of embryos), regulate the feeding practices and thereby indirectly influence milk quality. These variables are modeled as exogenous covariates that influence feeding practice indicators.

Empirical and review literature demonstrates that inadequate milking practices and equipment hygiene [85], along with a heightened incidence of mastitis (indicated by elevated somatic cell count), are associated with diminished lactose levels and fluctuating, frequently reduced fat and modified protein content (specifically casein hydrolysis), as well as decreased solid non-fat (SNF) in goat milk [86,87]. These variables were considered potential biological and management covariates because they may contribute to variation in milk composition independently of feeding-management practices.

### 2.4. Physiological Variables

Age, body weight, milk yield, and stage of lactation are closely linked traits that shape milk production and composition in goats. Milk yield typically increases after the first kidding, reaches a peak in mature animals, and then gradually declines [88]. These age-related changes are usually accompanied by an increase in body weight and metabolic capacity, which facilitate the synthesis of milk fat and protein and the ingestion of more feed, provided that energy balance is sufficient [89]. Research has demonstrated that older, multiparous goats tend to produce more milk [90] and often higher levels of fat and protein [91], although the strength of these effects varies by breed and management system [11,16].

Low body weight or poor body condition is known to diminish milk fat and protein content as a result of inadequate energy reserves, while lactose is generally less affected unless energy deficiency is severe [92,93]. Body weight was considered an important biological variable but could not be measured because data collection involved single farm visits across numerous farms that lacked standardized weighing equipment. As a result, including body weight in the analysis was not feasible.

To address this limitation, age and annual milk yield were used as practical proxies for underlying biological processes. Age functions as a practical proxy for physiological maturity, rumen development, and parity effects, while annual yield reflects productive capacity and nutrient partitioning toward lactation. Farmers frequently adjust nutritional management strategies based on these interconnected traits, emphasizing the importance of age, body weight, and production history in their feeding practices. For instance, feeding intensity, concentrate allocation, and energy supplementation are adapted according to the goats’ physiological status [69,83,94]. 

Goat age, annual milk yield [35,88], lactation period [11,16], and mastitis frequency [86] were retained in all candidate SEMs as a priori biological covariates because they are well-established determinants of milk composition and represent potential confounders of the associations between feeding-management practices and milk quality. Accordingly, only the feeding-management variables were subjected to the predictor-selection procedure described below, whereas these biological covariates were retained in all alternative model specifications.

## 3. Materials and Methods

### 3.1. Study Design and Data Collection

The research was conducted in Central Macedonia, Northern Greece, an area characterized by a temperate climate and a long tradition of small-ruminant dairy production. The study focused exclusively on farms rearing the indigenous Greek goat breed under semi-intensive management systems because this combination represents the predominant production model and the most economically important form of regional goat husbandry. Semi-intensive goat production in Central Macedonia combines daily grazing on natural or cultivated pastures with strategic indoor supplementation using hay, concentrates, mineral-vitamin mixtures, and other nutritional supplements according to animal requirements and feed availability [51]. Animals are generally housed overnight or during adverse weather conditions, while health management, reproduction, and milking practices remain under the direct supervision of the farmer. This production system is a middle ground between extensive grazing and fully intensive indoor production. It is the most common way to manage indigenous Greek dairy goats in the region [95].

The indigenous Greek goat was selected because of its ecological adaptability, resilience to local forage conditions, and ability to produce consistently high-quality milk under moderate-input feeding systems. The semi-intensive production system was selected because it combines controlled supplementary feeding with daily grazing, thereby providing a balanced representation of both concentrate- and pasture-based nutritional inputs, which are central to the objectives of the present study.

To capture the natural variation in feeding regimes throughout the production cycle, milk sampling was organized across the three biologically and management-defined lactation periods followed in the region. Due to the typical seasonal breeding pattern-kidding mainly between December and February-farms were approached during (i) early lactation (January–March), (ii) mid-lactation (April–June), and (iii) late lactation (July–September). This ensured that farms were sampled when goats were naturally in the corresponding lactation stage, making comparisons across early, mid-, and late lactation directly attributable to on-farm feeding practices.

A total of 242 semi-intensive farms were recruited through convenience sampling because no comprehensive regional sampling frame of eligible farms was available and participation depended on farmers’ willingness to provide milk samples and management information. Each participating farm provided a single milk sample from one lactating goat and filled out a standardized questionnaire to collect information regarding feeding practices, animal characteristics, and management. One lactating goat was sampled from each participating farm because the primary objective of the study was to investigate associations between farm-level feeding management practices and milk quality rather than to estimate within-farm animal variability. Since feeding practices, supplementation strategies, housing conditions, and general management are largely shared among goats within the same farm, sampling multiple animals would have produced correlated observations requiring hierarchical or multilevel modeling. By selecting one goat per farm, each milk sample was associated with a distinct farm-management system, thereby maximizing the diversity of feeding practices included in the survey while avoiding within-farm dependence among observations. Accordingly, the farm, rather than the individual goat, constituted the primary observational unit because the explanatory variables represented farm-level management decisions. Although this design does not permit evaluation of within-farm variability in milk composition, it is well suited to investigate associations between management practices and milk composition across commercial semi-intensive goat farms. Accordingly, no additional restrictions regarding parity, production level, or body weight were imposed before sampling because the objective was to characterize routine commercial management conditions rather than standardized experimental animals. Instead, age, annual milk yield, and lactation stage were recorded and subsequently incorporated into the statistical analyses as covariates or control variables where appropriate. Farms in which parturition had occurred fewer than seven days before sampling were excluded to avoid the collection of colostrum.

Given that the sample involved 242 different goats from 242 farms, individual weighing was not feasible during single-visit sampling due to logistical constraints associated with single-visit sampling across dispersed farms, lack of weighing infrastructure, and the need to avoid imposing handling stress on animals during routine milking. Because body weight was not measured, the analyses relied on directly recorded physiological characteristics, including age and annual milk yield, which partially capture differences in physiological maturity and productive capacity.

This omission is methodologically justified due to the following reasons: (i) age (or age category) and weight are usually closely correlated in dairy goats [96,97], indicating that age can effectively serve as a proxy for body size as an indicator of physiological maturity; (ii) annual milk production partially reflects metabolic capacity associated with greater body reserves [92]; and (iii) the exclusion of a highly correlated yet unmeasurable variable reduces the risks of model misspecification.

### 3.2. Milk Sampling and Laboratory Analysis

Milk samples were obtained directly from individual goats during afternoon milking sessions corresponding to each lactation period. Approximately 60 mL of milk was taken using volumetric flasks and then transferred to vials containing sodium azide as a preservative. These samples were promptly placed in a plastic refrigerator with ice packs to maintain their integrity. For a duration of 15–16 h, the samples were stored at a temperature of 4 °C. The analysis of milk composition for fat, protein, lactose, and total solids was performed using the MilkoScan™ 4000 infrared milk analyzer (FOSS, Hillerød, Denmark).

### 3.3. Questionnaire Distribution and Data Collection

A structured questionnaire was administered to all 242 participating farmers at the time of milk sampling to collect information on nutritional management practices during each lactation stage. Prior to the main survey, a pretest of the questionnaire was done with 10 farmers to assess its clarity and applicability.

The questionnaire was structured to collect information on nutritional management practices implemented throughout the production cycle of dairy goats. It included questions on forage resources and roughage types, estimated pasture intake, concentrate use, concentrate type and daily allocation, criteria used to determine concentrate allowance, nutritional strategies adopted during late pregnancy to ensure adequate energy intake, and feeding adjustments implemented as milk production declined during mid lactation. Additional questions recorded management and health information, including the frequency of mastitis, whereas goat-level production characteristics, including the age of the sampled goat and annual milk yield, were recorded separately. Most questionnaire items were closed-ended and were recorded as binary (yes/no) responses or predefined categorical variables, allowing their subsequent coding as candidate feeding-management variables for SEM analysis.

Farmers reported whether specific feeding practices or supplements (e.g., energy-rich feeds, protein sources, vitamin/mineral supplementation) were routinely applied during each of the three lactation stages (early, mid-, and late lactation), irrespective of the lactation stage of the goat sampled at the time of the farm visit. Thus, every participating farmer provided information on feeding management for all three lactation stages based on the routine practices implemented on the farm. Accordingly, the feeding-practice variables represent farm-level management strategies implemented throughout the lactation cycle, whereas milk composition was measured from the sampled goat at its current lactation stage.

Responses were coded separately for each lactation stage in binary form (1 = practiced, 0 = not practiced) and subsequently linked to the corresponding milk sample. Additional items captured physiological and management characteristics, as well as goat-level information, including the age and annual milk yield of the sampled goat. Informed consent was obtained from all farm owners prior to participation.

Because the questionnaire was administered through face-to-face interviews and reviewed for completeness before the interview was concluded, no missing responses were recorded. Consequently, the final dataset comprised complete observations for all 242 farms; no cases were excluded because of missing data, and no missing-data handling procedures (e.g., listwise deletion, Full Information Maximum Likelihood, or multiple imputation) were required.

### 3.4. Statistical Framework and Analysis

A hybrid structural equation model (SEM) was constructed, in which milk quality was specified as a latent construct measured by the log-transformed percentages of protein, fat, lactose, and solids-not-fat. Feeding-management practices together with goat age, lactation period, and mastitis frequency, were incorporated as observed exogenous variables according to the hypothesized conceptual framework. Annual milk yield was modeled as an endogenous observed variable because it was hypothesized to be influenced by goat age while simultaneously contributing to variation in the latent milk-quality construct.

To identify the latent milk-quality construct, the loading of milk protein was fixed to unity. Milk protein was selected as the reference indicator because preliminary analyses indicated that it provided the strongest and most stable standardized loading among the observed indicators.

Management variables and physiological characteristics were incorporated into the SEM as covariates because they represent established sources of biological and management-related variation that may confound the associations between feeding-management practices and milk quality.

Because the explanatory variables describe routine farm-level feeding strategies implemented during the different lactation stages rather than the exact ration consumed by the sampled goat on the day of milk collection, the SEM should be interpreted as evaluating associations between management practices and milk composition under commercial production conditions rather than short-term physiological responses to specific feeding events.

Several feeding-practice variables were recorded separately for different lactation stages. Prior to SEM estimation, repeated binary variables were examined for consistency across lactation stages using the Phi coefficient. Aggregation was applied selectively and only to feeding practices that demonstrated strong consistency across lactation stages. Feeding variables showing meaningful stage-specific variation were retained as separate predictors because they reflected biologically distinct nutritional management during different phases of lactation.

Variables exhibiting strong between-stage associations (approximately Φ ≥ 0.70) were considered to represent stable farm-management practices rather than independent stage-specific decisions and were therefore aggregated into a single binary indicator to enhance model parsimony and reduce redundancy among highly associated predictors. The observed Phi coefficients ranged from 0.745 to 0.856 for the aggregated variables (Table 1), indicating substantial consistency in farmers’ feeding strategies across lactation. Specifically, Phi coefficients were 0.856, 0.821, and 0.745 for the dairy mix with vitamins and minerals across lactation stages, 0.803 for concentrate feeding (300–500 g/day) between mid and late lactation, and 0.752 for protein concentrate supplementation between early and late lactation (all *p* < 0.001).

Prior to SEM estimation, a confirmatory factor analysis (CFA) was performed to assess the hypothesized measurement model of goat milk quality. The convergent validity of the latent construct was assessed using Average Variance Extracted (AVE), whereas internal consistency was evaluated using Composite Reliability (CR). Values of AVE ≥ 0.50 and CR ≥ 0.70 were considered indicative of acceptable convergent validity and construct reliability, respectively [98,99]. Fornell & Larcker (1981) [98] explicitly state that if CR exceeds 0.60, convergent validity may still be considered adequate even when AVE is below 0.50, particularly when the construct has strong theoretical support.

Although several feeding-practice variables were binary, they were specified exclusively as observed exogenous predictors rather than as indicators of latent constructs. The latent milk-quality construct was measured solely by continuous variables (protein, fat, lactose, and solid non-fat), all of which were logarithmically transformed prior to analysis. Consequently, maximum-likelihood (ML) estimation was considered appropriate because the principal distributional assumptions relate primarily to the continuous endogenous variables rather than the binary exogenous predictors. To further evaluate the robustness of the ML estimates against potential departures from multivariate normality, non-parametric bootstrap standard errors, 95% bias-corrected confidence intervals, and the Bollen–Stine bootstrap test were estimated using 5000 bootstrap resamples in AMOS.

The available sample size (*n* = 242) was considered adequate for estimation of the proposed SEM because the model comprised a single latent construct measured by four observed indicators together with a relatively limited structural component. Consistent with current methodological recommendations, sample-size adequacy was evaluated primarily with respect to model complexity, parameter estimation, and overall model fit rather than according to universal observations-to-parameter rules [100,101]. Results with *p* < 0.05 were considered statistically significant. The overall fit of the model was evaluated using the χ^2^/df ratio (CMIN/DF), the Comparative Fit Index (CFI), the Incremental Fit Index (IFI), the Tucker–Lewis Index (TLI), the Root Mean Square Error of Approximation (RMSEA), and the Standardized Root Mean Square Residual (SRMR). Conventionally, χ^2^/df values < 3, CFI/IFI/TLI values ≥ 0.90, RMSEA values ≤ 0.08, and SRMR values < 0.08 indicate acceptable model fit [100,102]. Following estimation of the hypothesized model, modification indices were examined to identify potential areas of localized model misfit. The standardized residual covariance matrix was also examined to identify localized areas of model misfit. Residuals exceeding the recommended thresholds (approximately |1.96| or, more conservatively, |2.58|) were inspected to evaluate potential model misspecification before any theoretically justified model refinements were considered [100]. Analyses were performed in SPSS (V. 26) and AMOS (V.26).

#### Statistical Assumptions

Assessment of SEM assumptions. Prior to maximum likelihood estimation, the assumptions underlying SEM were evaluated. Univariate normality of the continuous variables was assessed through the application of skewness and kurtosis statistics subsequent to logarithmic transformation. Multivariate normality was assessed through the application of Mardia’s multivariate kurtosis coefficient along with its corresponding critical ratio. Multicollinearity among the exogenous feeding-practice variables was assessed using tolerance and variance inflation factor (VIF) statistics. Potential multivariate outliers were examined using Mahalanobis distance. As described above, bootstrap procedures were used to assess the robustness of the parameter estimates and the overall model fit. Within the assumption diagnostics, the Bollen–Stine bootstrap was interpreted together with the remaining normality and multicollinearity diagnostics. The results of the assumption diagnostics and bootstrap analyses are summarized in Table 2.

As shown in Table 2, the assessment indicated no serious violation of the assumptions required for maximum likelihood estimation. Although several observed feeding-practice variables were binary, they were specified as exogenous predictors rather than indicators of latent constructs. The multivariate normality assessment showed only a mild departure from normality (critical ratio = 2.706), while multicollinearity among predictors was negligible (maximum VIF = 1.30). Screening based on Mahalanobis distance did not identify observations warranting exclusion. Bootstrap standard errors and 95% bias-corrected confidence intervals showed negligible differences from the corresponding maximum-likelihood estimates, indicating stable parameter estimation under repeated resampling. Furthermore, the Bollen–Stine bootstrap (5000 resamples) yielded a non-significant result (*p* = 0.054), supporting the robustness of the maximum-likelihood solution.

Following estimation of the hypothesized model, modification indices were inspected to identify localized areas of model misfit. Only modifications supported by biological knowledge of goat feeding management and resulting in improved model fit without altering the substantive interpretation of the hypothesized model were retained.

Assessment of prefecture-level clustering: Because one goat was sampled from each farm, within-farm variability could not be estimated. Nevertheless, farms were nested within seven prefectures. To evaluate whether regional clustering could influence the SEM estimates, intercept-only linear mixed-effects models were fitted separately for each continuous milk-quality indicator (LnPROT, LnFAT, LnLACT, and LnSNF), with prefecture specified as a random intercept. Intraclass correlation coefficients (ICCs) were computed from the estimated variance components to quantify the proportion of total variance attributable to differences between prefectures. The results of the prefecture-level clustering analysis are presented in Table 3.

As shown in Table 3, the estimated between-prefecture variance was negligible for all four milk-quality indicators, resulting in intraclass correlation coefficients that were effectively zero. These findings indicate that geographical clustering at the prefecture level contributed minimally to the overall variability in milk composition. Consequently, prefecture-level clustering was considered unlikely to influence the SEM parameter estimates or their standard errors, supporting the use of a single-level structural equation model.

## 4. Results

### 4.1. Descriptive Analysis

According to farmer reports, the average annual milk production per goat was 177 ± 64 kg per lactation, with a maximum of 456 kg and a minimum of 50 kg. The average age of the goats in the sample was 3.2 ± 1.1 years, ranging from 1.5 to 5.8 years. Across all sampled goats (*n* = 242), milk contained on average, 4.81% fat (SD = 0.74), 3.82% protein (SD = 0.35), 4.44% lactose (SD = 0.35), and 9.19% solid non-fat (SNF) (SD = 0.61). When examined by lactation stage, milk from goats sampled during early lactation (*n* = 73) contained 5.03% fat (±0.82), 3.87% protein (±0.37), and the highest lactose concentration (4.6 3% ± 0.17). The mean SNF content was 9.42% (±0.51). During mid-lactation (*n* = 88), milk composition showed the lowest values for most components: fat averaged 4.45% (±0.62), protein 3.65% (±0.29), lactose 4.26% (±0.27), and SNF 8.83% (±0.56). During late lactation (*n* = 81), fat levels increased again (5.00% ± 0.63), protein content reached its highest mean value (3.96% ± 0.32), and SNF averaged 9.39% (±0.56). Lactose content (4.48% ± 0.44) was intermediate between that observed during early and mid-lactation.

Of the 26 feeding practices initially recorded, seven variables (aggregated where appropriate) were retained in the final structural equation model as predictors of goat milk quality, following the predefined model-selection procedure. Table 4 presents the frequencies of the feeding-practice variables retained in the final model. These frequencies represent the number of farms reporting the use of each feeding practice during the specified lactation stage and are therefore based on questionnaire responses from all 242 participating farmers rather than only the goats sampled during that particular stage.

According to the questionnaire responses, 50.4% of farms reported providing concentrated feeding at a rate of 300–500 g/day, aggregated across mid- and late-lactation periods. The provision of a dairy mix containing vitamins and minerals, aggregated across all three lactation periods, was more common, with 69.0% of goats receiving supplementation. Protein concentrate supplementation in early and late lactation was rare, at 21.1%. The first and mid-lactation stages had similar pasture consumption of 3–4 kg/day (45.5% and 47.9%). Mid-lactation grass and legume hay was consumed by 38.0% of goats, whereas in late lactation goat balancers were consumed by 23.1% of goats. The descriptive analysis indicates that mineral supplementation was the predominant feeding practice throughout lactation stages, whereas protein supplementation and the use of a goat balancer were the least prevalent.

### 4.2. CFA Results

**Figure 1:**
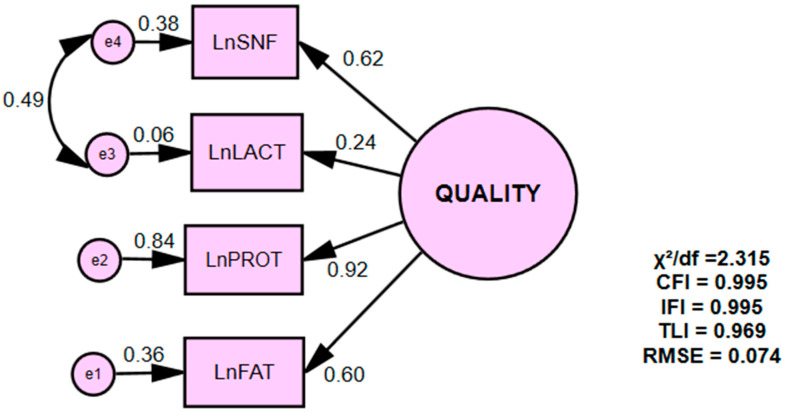
CFA model of milk quality.

Figure 1 shows the CFA model for the quality of goat milk together with the standardized regression weights and fit indices. The measurement model demonstrated a satisfactory overall fit to the data (CMIN/DF = 2.315; CFI = 0.995; IFI = 0.995; TLI = 0.969; RMSEA = 0.074).

All four observed indicators-log-transformed protein, fat, lactose, and solid non-fat-loaded significantly (*p* < 0.001) and positively on the latent construct of milk quality. In unstandardized terms, loadings ranged from 2.064 (LnSNF) to 4.633 (LnFAT). Standardized factor loadings showed that protein (β = 0.916) was the strongest indicator of milk quality, followed by solid non-fat (β = 0.620), fat (β = 0.601), and lactose (β = 0.244). Thus, protein contributed most strongly to the latent factor, whereas lactose had a weaker but still significant relationship.

The Composite Reliability was 0.706, exceeding the recommended threshold of 0.70 and indicating acceptable internal consistency. The AVE was 0.411, which was below the conventional threshold of 0.50. This lower AVE was primarily attributable to the relatively weak standardized loading of lactose (λ = 0.244). Nevertheless, according to Fornell and Larcker, convergent validity may still be considered acceptable when Composite Reliability exceeds 0.60 despite an AVE below 0.50. Despite the relatively low loading of lactose, the latent construct demonstrated acceptable convergent validity because Composite Reliability exceeded the recommended threshold and three of the four indicators exhibited strong and statistically significant factor loadings.

### 4.3. SEM Results

**Figure 2:**
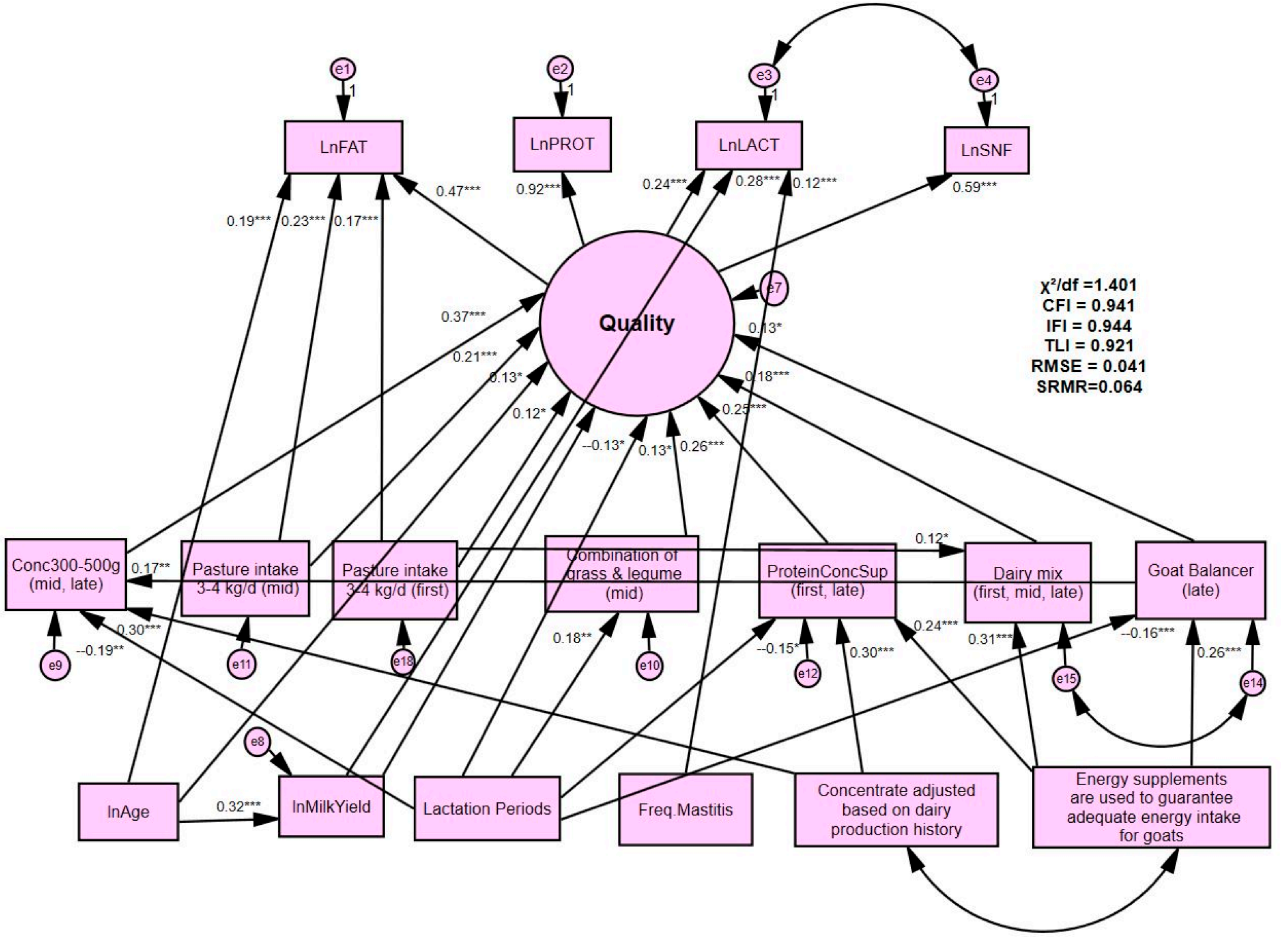
Final SEM illustrating the associations among feeding-management practices, management covariates, and goat milk quality. Asterisks denote statistical significance (* *p* < 0.05, ** *p* < 0.01, *** *p* < 0.001).

The structural equation model = evaluating the associations between feeding management practices and goat milk quality exhibited a satisfactory overall fit (CMIN/DF = 1.401; CFI = 0.941; IFI = 0.944; TLI = 0.921; RMSEA = 0.041; SRMR = 0.064). Examination of the standardized residual covariance matrix revealed no systematic pattern of localized model misspecification. Only two standardized residuals slightly exceeded the conservative threshold of |2.58|, whereas the remaining residuals were below this value, supporting the adequacy of the final model specification. The squared multiple correlation for the latent variable of milk quality was 0.466, signifying that almost 47% of the variance in milk quality was elucidated by the model. The overall structure of the final SEM, including the retained structural paths, latent construct, observed indicators, and standardized regression coefficients, is presented in Figure 2.

As described in Section 3, three alternative SEMs were estimated to evaluate the robustness of the predictor-selection procedure (Table 5). Model 1 included all 12-candidate feeding-management predictors identified from the questionnaire. Model 2 retained the nine predictors that demonstrated the strongest combination of statistical support, biological plausibility, and unique explanatory contribution. Model 3 represented the final parsimonious model, retaining seven predictors after considering statistical significance, biological plausibility, overlap in the information explained by related feeding-management variables, multicollinearity, and overall model fit. As shown in Table 5, progressive model refinement substantially improved overall model fit while reducing model complexity, supporting the selection of the final parsimonious model.

The regression weights and standardized path coefficients of the selected final structural equation model are presented in Table 6. Several feeding-management practices showed statistically significant positive associations with the latent construct of milk quality.

The latent construct quality was most strongly represented by milk protein (standardized loading = 0.922), followed by solid non-fat (0.589), fat (0.467), and lactose (0.242), with all factor loadings being highly significant (Table 6). Among the feeding-management practices, providing 300–500 g/day of concentrate during mid- and late lactation exhibited the strongest positive association with quality (β = 0.369, *p* < 0.001). Positive associations were also observed for the combination of grass and legume forage during mid lactation (β = 0.263, *p* < 0.001), protein concentrate supplementation during early and late lactation (β = 0.252, *p* < 0.001), pasture intake of 3–4 kg/day during mid lactation (β = 0.213, *p* < 0.001), dairy mix supplementation (β = 0.181, *p* = 0.001), pasture intake during early lactation (β = 0.125, *p* = 0.021), and goat balancer use during late lactation (β = 0.126, *p* = 0.026). In contrast, annual milk yield was negatively associated with quality (β = −0.129, *p* = 0.021). Pasture intake of 3–4 kg/day during both early and mid-lactation was additionally associated with higher milk fat content (β = 0.171 and β = 0.230, respectively; both *p* < 0.001), whereas annual milk yield was positively associated with lactose concentration (β = 0.281, *p* < 0.001). The two retained structural paths indicated that goat balancer use during late lactation was positively associated with the provision of 300–500 g/day concentrate during mid and late lactation (β = 0.169, *p* = 0.005), while pasture intake of 3–4 kg/day during early lactation was positively associated with the use of a dairy mix containing vitamins and minerals (β = 0.122, *p* = 0.042). These relationships are biologically plausible and improved overall model fit without altering the substantive interpretation of the structural model.

Table 7 summarizes the relationships between the management covariates and the feeding-management practices included in the SEM. Adjusting the quantity of cereals and concentrates according to the goat’s dairy production history was positively associated with the provision of 300–500 g/day of concentrate during mid- and late lactation (β = 0.301, *p* < 0.001) and protein concentrate supplementation during early and late lactation (β = 0.300, *p* < 0.001). Likewise, the use of energy supplements during late pregnancy was positively associated with subsequent dairy mix supplementation (β = 0.308, *p* < 0.001), goat balancer use (β = 0.263, *p* < 0.001), and protein concentrate supplementation (β = 0.244, *p* < 0.001). Increasing age was positively associated with annual milk yield (β = 0.315, *p* < 0.001), milk fat content (β = 0.191, *p* < 0.001), and the latent construct of milk quality (β = 0.126, *p* = 0.026). The lactation period was positively associated with milk quality (β = 0.133, *p* = 0.018) and the use of a grass–legume forage combination (β = 0.181, *p* = 0.004) but negatively associated with the provision of 300–500 g/day concentrate (β = −0.186, *p* = 0.002), protein concentrate supplementation (β = −0.145, *p* = 0.012), and goat balancer use (β = −0.162, *p* = 0.007). Finally, mastitis frequency showed a significant and negative association with milk lactose concentration (β = −0.122, *p* = 0.013).

Significant correlations were found between covariates and error terms. Production-based concentrate allocation correlated with late-gestation energy supplementation (r = 0.156, *p* = 0.016), reflecting intensified feeding for high-requirement animals. A residual correlation between the goat balancer (late lactation) and the vitamin–mineral mix (r = 0.168, *p* = 0.015) indicated shared supplementation practices. The strong lactose-SNF error correlation (r = 0.573, *p* < 0.001) was physiologically expected because lactose is the primary determinant of SNF.

Table 8 shows the calculated percentage changes in milk components generated by unstandardized total effects (direct plus indirect effects) extracted from AMOS. For binary feeding practices, percentage changes were calculated as 100 × [exp (β) − 1] and represent the proportional change in each milk component associated with the presence versus absence of the respective feeding practice. In contrast, milk yield and age were modeled as continuous log-transformed variables; therefore, their unstandardized coefficients are interpreted directly as elasticities and are reported without transformation.

Across all feeding practices, milk protein and fat exhibited the largest proportional associations, whereas lactose consistently showed the smallest relative changes. Concentrate supplementation at 300–500 g/day during mid- and late lactation was associated with the largest proportional increase in milk protein (5.97%), together with higher fat (5.13%), SNF (2.84%), and lactose (1.51%). Protein concentrates supplementation and the provision of a grass–legume forage mixture during mid-lactation were likewise associated with higher protein and fat contents. Pasture intake of 3–4 kg/day was primarily associated with milk fat, particularly during mid-lactation, where the estimated proportional increase reached approximately 10.2%.

The elasticity estimates indicate that a 1% increase in milk yield is associated with reductions of 0.009% in protein, 0.008% in fat, and 0.004% in SNF, while lactose increases by 0.018%. These associations are consistent with the physiological dilution effect, whereby increasing milk volume reduces the concentration of milk solids without necessarily implying a reduction in the absolute synthesis of milk constituents. A 10% increase in age (equivalent to approximately 3.8 months at the sample mean age of 3.2 years) was associated with increases of 0.19% in protein, 0.93% in fat, 0.09% in SNF, and 0.25% in lactose, with fat exhibiting the strongest relative response.

## 5. Discussion

The results indicate that the quality of the goat’s milk is a composite reflection of protein, fat, lactose, and solid non-fat (SNF) content during the early, mid, and late stages of lactation. Milk protein exhibited the strongest standardized loading, confirming its dominant contribution to the latent milk quality construct and its established importance for both the nutritional value of goat milk and cheese-making performance. Fat and SNF also contributed substantially, consistent with their recognized roles in determining milk energy content, compositional density, processing characteristics, and commercial milk quality assessment [50,52,53]. Although lactose exhibited a comparatively lower standardized loading (β = 0.244), this finding is biologically plausible because lactose concentration is tightly regulated by mammary gland physiology through its central role in osmotic regulation and milk secretion, resulting in considerably lower biological variability than fat and protein across breeds, lactation stages, and production systems [46,47,48]. Previous studies have likewise reported relatively small variation in lactose concentration compared with the other principal milk constituents [34,35]. Consequently, lactose is expected to contribute less to the between-animal variation captured by the latent construct Quality, despite remaining one of the principal compositional constituents routinely used to characterize goat milk quality [3]. For this reason, lactose was retained in the measurement model to preserve the biological completeness and conceptual validity of the latent construct.

The correlated residual between lactose and SNF reflects their well-established compositional relationship rather than a misspecification of the measurement model. Specifically, SNF comprises lactose together with proteins, minerals, vitamins, and other dissolved solids and is widely recognized as a comprehensive indicator of milk compositional integrity, nutritional density, and technological quality [50,56]. Consequently, some residual covariance between lactose and SNF is expected independently of their common association with the latent quality construct. Modeling this covariance therefore reflects the known biochemical composition of milk and enables the latent construct to represent the multidimensional concept of goat milk quality as routinely applied in dairy science and commercial quality evaluation [3,55].

Overall CFA results support the concept of the quality of milk as a complex latent variable and the potential inclusion of it in a model to analyze the effects of management and nutrition.

The SEM analysis identified concentrate feeding (300–500 g/day) during mid- and late lactation as the strongest feeding-management predictor associated with the latent milk-quality construct. At the component level, this practice was associated with higher protein by 5.97%, fat by 5.13%, SNF by 2.84%, and lactose by 1.51%, quantitatively confirming that its strong latent effect (β = 0.369) is primarily transmitted through improvements in milk solids. This method, adopted by 46.3% of producers in the sample, may represent a balanced and cost-effective feeding strategy associated with improved milk composition under the management conditions represented in this study. A biologically plausible explanation is that energy-dense concentrates increase fermentable carbohydrate supply, supporting microbial protein synthesis and mammary substrate availability. Many feeding trials with goats have shown that adding more concentrate to their diet increases production and often raises fat and protein levels (or their yields). This effect is partly because it helps with energy balance and ruminal volatile fatty acid profiles. These mechanisms are confirmed in the positive path (β = 0.369) [13,64].

The use of grass and legume hay mix at mid-lactation had a positive effect (β = 0.263) on milk quality, increasing protein (4.40%), fat (3.77%), solids-not-fat (2.02%) and lactose (1.11%). These results are consistent with previous findings that mixed swards improve milk quality by higher protein content and digestibility from legumes, which improve fat content and quality with better forages [66].

Among the eight types of concentrate supplementation, three had a statistically significant positive effect on milk quality. The significant positive effect (β = 0.252) of protein concentrate during early and late lactation is probably due to the increased protein requirements in stages of negative energy balance and reduced feed intake. No effect was found at mid-lactation when basal feeds may have been sufficed. This pattern is consistent with findings by Fu et al. [7], where lower protein diets had a more pronounced negative effect early in lactation, but the effects lessened over time. Protein concentrate supplementation was associated with approximately 5.0% higher protein and 4.3% higher fat concentrations, confirming its role in supporting amino acid supply during periods of negative energy balance.

A dairy blend fortified with vitamins and minerals was positively associated with the latent milk-quality construct at all stages (β = 0.181). Its effects were moderate but consistent on all components (protein +3.15%, fat +2.74%). This is in agreement with other studies which have shown that supplementing the diet with trace elements and vitamins improve milk composition, yield and health at all stages [78,79]. The goat balancer was significant only in late lactation, being associated with approximately 3.6% higher protein and 3.2% higher fat concentrations, together with smaller increases in SNF (1.7%) and lactose (0.9%).

Annual milk yield was negatively associated with the latent milk-quality construct (β = −0.129). Specifically, a 1% increase in annual milk yield was associated with reductions of approximately 0.009% in protein, 0.008% in fat, and 0.004% in solid non-fat (SNF), whereas lactose increased by approximately 0.018%. These findings are more appropriately interpreted as reflecting the well-recognized physiological dilution effect rather than a true biological antagonism between milk yield and milk quality. As milk secretion increases, the greater volume of the aqueous phase dilutes the relative concentrations of milk solids, even though the absolute synthesis of protein and fat by the mammary gland may remain unchanged or even increase. Consequently, the observed negative associations describe changes in milk composition (concentrations) rather than reductions in total nutrient production. Similar dilution effects have been widely reported in dairy ruminants and represent a normal physiological consequence of increased milk production rather than impaired mammary function [10,46].

Age showed a modest positive association with the latent milk quality construct (β = 0.126) and fat content (β = 0.191). Older goats produced milk with significantly higher fat content and a moderate increase in lactose and protein, a result aligned with the findings of Garcia-Olarte et al. [91]. The significant positive impact of age on annual milk yield (β = 0.315) is consistent with established lactation-curve dynamics in which production improves after first parity and peaks in mature animals [88,90]. It is important to acknowledge that outcomes are not uniform across all breeds or management systems [11,16].

Lactation period was retained as a biological covariate and was associated both directly with the latent milk-quality construct and indirectly through several feeding-management practices. It negatively affects the provision of concentrates (300–500 g), protein concentrate supplementation, and goat balancer, so their use diminishes as lactation advances. This trend likely reflects economic management decisions alongside the natural decline in milk yield and metabolic demands during late lactation [9,15].

The positive influence of the lactation period on the combination of grass and legume hay implies that there will be a greater reliance on grass–legume hay during the later phases of lactation. This pattern is consistent with practical feeding management because many farmers gradually shift toward more forage-based diets (alfalfa, clover, vetch, etc.) to reduce costs of feed and maintain body condition. The use of mixed hays comprising grasses and legumes can increase digestible energy supply and improve nitrogen use efficiency, promoting prolonged milk component synthesis without excessive concentrate feeding [63].

The lactation stage was positively associated with the quality of milk and shaped specific practices (less concentrate/protein/balancer and more mixed hay later). These paths are coherent with practical management: as lactation advances and intake stabilizes, farms often rebalance rations (less purchased concentrate, more forage), while milk composition naturally fluctuates by stage because of energy balance and hormonal milieu. These adjustments align with established evidence identifying the lactation stage as a primary determinant of goat milk composition [3]. These findings align with experimental evidence demonstrating that dietary forage–concentrate balance interacts with lactation stage to influence milk yield and compositional traits, highlighting the importance of adaptive nutritional management throughout the lactation curve [64].

Finally, the model shows that frequent mastitis occurrences result in a decrease in lactose concentration in milk, corroborating the findings of other investigations [86,87].

The present study was designed to evaluate associations between farm-level feeding-management practices and goat milk quality within an integrated structural equation model rather than to compare feeding effects across individual lactation stages. Although feeding practices were recorded separately for early, mid-, and late lactation and lactation stage was incorporated as a biological covariate, the objective was to estimate their overall associations with the latent milk-quality construct while accounting for physiological variation across lactation. Consequently, differences among lactation stages should be interpreted descriptively rather than as direct comparisons of feeding effects between stages.

## 6. Implications

Overall, the observed feeding-management practices were more strongly associated with milk composition than with variation in annual milk yield, suggesting that the management strategies implemented on the participating farms were compatible with maintaining favorable concentrations of milk solids under semi-intensive production conditions. Although the observational design does not permit inference regarding farmers’ motivations or decision-making processes, the identified associations indicate that stage-specific feeding management emphasizing moderate concentrate supplementation, high-quality forage, and targeted nutritional supplementation is compatible with improved milk composition while maintaining commercial production.

The research further validates that nutritional requirements vary considerably throughout the stages of lactation, necessitating strategic management. As milk yield decreases during late lactation, the results suggest that reducing concentrate allocation during late lactation may help avoid metabolic inefficiency and prevent over-conditioning. The integration of mixed grass–legume hay, such as alfalfa, clover, and vetch, into goat diets may enhance digestible energy and nitrogen-use efficiency while reducing reliance on concentrate feeds. This is crucial for enhancing feed resource efficiency, particularly in Mediterranean and semi-arid areas where dependence on purchased feeds increases production expenses and market vulnerability.

Improving on-farm forage quality enables producers to enhance milk solids, reduce nitrogen losses, and decrease emission intensity per product unit, thereby strengthening environmental performance. From an economic perspective, the findings suggest that feeding management strategies associated with improved milk composition-particularly protein and fat-may provide greater value than approaches focusing exclusively on increasing milk volume. Previous studies have similarly demonstrated that production systems and seasonal feeding practices influence the compositional quality and nutritional characteristics of goat milk, highlighting the potential of nutritional management to improve milk properties relevant to dairy processing [51].

Most small- and medium-sized goat farms in Central Macedonia sell raw milk directly to dairy processors and cheese makers instead of processing it on the farm [95]. Because the commercial value of goat milk is closely linked to its technological suitability for cheese production, improvements in milk solids may enhance cheese yield, processing efficiency, and the overall value of the raw milk supplied to the dairy industry. Although some farms produce artisanal dairy products, on-farm milk processing represents only a small proportion of regional production. Consequently, improving milk composition has practical economic importance primarily through increasing the quality and technological value of the milk delivered to processors rather than through on-farm cheese manufacture. These findings are therefore directly relevant to commercial goat farms operating under semi-intensive production systems in Greece [95] and in other Mediterranean regions where goat milk is predominantly marketed through the dairy processing sector.

At the policy and advisory level, these findings may inform the development of evidence-based nutritional management guidelines tailored to different stages of lactation. Promoting feeding strategies associated with improved milk composition, including appropriate concentrate allocation, high-quality forage utilization, and balanced nutritional supplementation, may contribute to more efficient use of feed resources while maintaining milk quality under commercial production conditions. Extension services can use these findings to develop stage-specific nutritional advisory programs that assist dairy goat producers in optimizing feeding decisions, improving milk composition, and enhancing the economic value of milk delivered to dairy processors.

## 7. Limitations and Future Research

Several caveats should be considered when interpreting the findings of this study. First, farms were recruited through convenience sampling across seven prefectures, and only one lactating goat was sampled from each participating farm. Although this design maximized the diversity of feeding management practices and avoided within-farm dependence among observations, the use of convenience sampling limits the external generalizability of the findings, while the observational study design precludes causal inference. Moreover, no additional restrictions regarding animal characteristics (e.g., parity, production level, or body weight) were imposed before sampling because the objective was to characterize routine commercial management conditions rather than standardized experimental animals. Age, annual milk yield, and lactation stage were recorded and incorporated into the statistical analyses where appropriate. Although age and annual milk yield were included as indicators of physiological maturity and productive capacity, body weight, body condition score, and parity of the sampled goat were not recorded. Consequently, age and milk yield could only partially account for differences in physiological maturity and metabolic status, and residual confounding associated with these unmeasured physiological characteristics, as well as breed, individual production potential, pasture botanical composition, or subclinical health conditions, cannot be completely excluded.

Feeding practices were recorded as routine management strategies reported by farmers for the different lactation stages rather than as contemporaneous measurements of the ration consumed by the sampled goat at milk collection. Consequently, the estimated associations should be interpreted at the farm-management level and should not be interpreted as establishing immediate cause-and-effect relationships between specific feeding events and milk composition. The selected categories reflect common feeding-management decisions under semi-intensive production systems and allow consistent data collection across a large number of commercial farms, but future studies based on precise measurements of feed intake and nutrient composition would provide more detailed quantitative estimates of these associations. In addition, several feeding-practice variables were aggregated across lactation stages because repeated measurements exhibited strong consistency. Although this approach reduced redundancy among highly correlated predictors and facilitated parsimonious SEM estimation, it may have obscured subtle stage-specific changes in feeding management.

Furthermore, the study was geographically confined to Central Macedonia, and extrapolation of the findings to other regions or production systems should therefore be undertaken with caution.

Finally, although structural equation modeling is well suited to evaluating theoretically specified relationships among observed and latent variables, the cross-sectional observational design adopted in the present study does not permit definitive causal inference because temporal precedence cannot be established and residual confounding cannot be completely excluded. Consequently, the estimated structural paths should be interpreted as statistical associations rather than causal effects. Longitudinal or randomized controlled feeding studies are required to establish causal relationships.

Future research should: (a) implement longitudinal or repeated-measures designs that follow the same animals throughout lactation; (b) quantify pasture nutritive value, dry matter intake, and concentrate consumption using continuous measurements; (c) include parity as an explicit grouping variable through multi-group SEM; (d) incorporate direct measurements of body weight, body condition score, and additional physiological indicators alongside milk yield to better account for animal-level heterogeneity; and (e) validate the present structural equation model in independent goat populations and alternative production systems to evaluate its generalizability. Such studies would facilitate more robust causal evaluation of the relationships between feeding-management practices and goat milk quality.

## 8. Conclusions

This study conceptualized goat milk quality as a multidimensional latent construct and applied structural equation modeling to examine its associations with feeding-management practices under commercial semi-intensive dairy goat production systems. The findings indicate that stage-specific feeding-management practices, particularly moderate concentrate supplementation, high-quality forage, targeted protein supplementation, and appropriate nutritional supplementation, were associated with more favorable milk composition, whereas increasing annual milk yield was associated with lower concentrations of protein, fat, and solids-not-fat. The latent-variable approach enabled the simultaneous evaluation of multiple interconnected feeding-management practices and biological covariates, providing a more integrated understanding of factors associated with goat milk composition than analyses based on individual milk components alone. The results further suggest that stage-specific nutritional management may help optimize milk composition while maintaining practical production objectives under commercial farming conditions.

Because one lactating goat was sampled from each participating farm, the findings should be interpreted as reflecting associations between farm-level management practices and milk composition rather than within-farm animal variation or causal effects. Nevertheless, by including a broad range of independent commercial farms, the study provides useful evidence regarding the relationships between routine feeding management and goat milk quality in semi-intensive production systems. Future longitudinal and controlled feeding studies incorporating repeated measurements from multiple animals within farms are warranted to confirm these associations, validate the proposed structural model, and establish causal relationships.

## Figures and Tables

**Table 1:** Associations between repeated feeding-practice variables used to justify aggregation.

Variable Aggregated	Lactation Stages Compared	Phi	*p*-Value
Dairy mix with vitamins and minerals	Early vs. Mid	0.856	<0.001
Dairy mix with vitamins and minerals	Early vs. Late	0.821	<0.001
Dairy mix with vitamins and minerals	Mid vs. Late	0.745	<0.001
Concentrate allocation (300–500 g/day)	Mid vs. Late	0.803	<0.001
Protein concentrate supplementation	Early vs. Late	0.752	<0.001

**Table 2:** SEM assumption diagnostics and bootstrap validation of parameter estimates.

Diagnostic	Result	Interpretation *
Absolute skewness	0.071–0.823	Acceptable (|skewness| < 2) [100]
Absolute kurtosis	0.074–1.519	Acceptable (|kurtosis| < 7) [100]
Mardia’s critical ratio **	2.706	No serious departure from multivariate normality (critical ratio < 5) [100,103]
Tolerance	0.769–0.959	No evidence of multicollinearity (tolerance > 0.20) [99]
Variance Inflation Factor (VIF)	1.04–1.30	Negligible multicollinearity (VIF < 5) [99]
Mahalanobis distance	Largest D^2^ = 42.65	No influential multivariate outliers were identified that warranted exclusion [100]
Bollen-Stine bootstrap	*p* = 0.054	Supports the robustness of the maximum-likelihood solution under bootstrap resampling [104]
SRMR	0.064	Acceptable residual fit (SRMR < 0.08) [102]

* Interpretation criteria were based on recommendations by Kline [100] for acceptable univariate and multivariate normality in SEM, Hair et al. [99] for multicollinearity diagnostics, and Bollen and Stine [104] for bootstrap assessment of model fit. Mahalanobis distance was used to screen for influential multivariate outliers, and all observations were retained because none showed evidence of undue influence on model estimation. ** The corresponding multivariate kurtosis coefficient was 8.842.

**Table 3:** Prefecture-level ICC analysis.

Outcome	Between-Prefecture Variance	Residual Variance	ICC	Interpretation
LnPROT	0.000	0.008208	≈0.000	Negligible clustering
LnFAT	0.000	0.023028	≈0.000	Negligible clustering
LnLACT	0.000	0.006492	≈0.000	Negligible clustering
LnSNF	0.000	0.004284	≈0.000	Negligible clustering

**Table 4:** Frequencies of farm-level feeding-management practices reported for each lactation stage (*n* = 242 farms).

Variable (Aggregated Where Applicable)	*n* (%) Used	*n* (%) Not Used	Notes
Concentrate feeding (300–500 g/day, mid & late lactation)	122 (50.4%)	120 (49.6%)	Aggregated across mid- and late lactation periods
Dairy mix with vitamins & minerals (first, mid & late lactation)	167 (69%)	75 (31%)	Aggregated across three lactation stages
Protein concentrates supplementation (first & late lactation)	51 (21.1%)	191 (78.9%)	Aggregated across first and late lactation periods
Pasture intake of 3–4 kilos daily (mid-lactation)	116 (47.9%)	126 (52.1%)	Mid-lactation
Combination of grass and legume hay (mid-lactation)	92 (38%)	150 (62%)	Mid-lactation
Goat balancer (late lactation)	56 (23.1%)	186 (76.9%)	Late-lactation
Pasture intake of 3–4 kilos daily (first lactation)	110 (45.5%)	132 (54.5%)	First-lactation

**Table 5:** Comparison of alternative SEM specifications used during predictor selection.

Model	Candidate Feeding Predictors	Predictors Retained	χ^2^/df	CFI	TLI	IFI	RMSEA	SRMR	AIC	BCC	Interpretation
Model 1	12	12	2.498	0.737	0.678	0.746	0.079	0.093	644.117	662.264	Exploratory model including all candidate predictors; inadequate overall fit despite significant paths.
Model 2	12	9	1.495	0.915	0.887	0.911	0.045	0.067	350.328	418.000	Improved model after removing predictors with limited unique explanatory contribution; acceptable fit but still less parsimonious than the final model.
Model 3 (Final)	12	7	1.401	0.941	0.921	0.944	0.041	0.064	278.857	289.835	Final parsimonious model with the best overall balance between model fit, biological interpretability, and simplicity.

**Table 6:** Regression weights and significance of the final structural equation model.

Affected Variables	Direction of the Effect	Observed Endogenous Variables	Estimate	S.E.	C.R	*p*
LnPROT	<---	Quality	1.000 (0.922) *			***
LnFAT	<---	Quality	0.867 (0.467)	0.120	7.234	***
LnSNF	<---	Quality	0.475 (0.589)	0.056	8.521	***
LnLACT	<---	Quality	0.252 (0.242)	0.069	3.651	***
Quality	<---	Concentrate provided: 300–500 g (mid & late)	0.058 (0.369)	0.009	6.590	***
Quality	<---	Pasture intake of 3–4 kg/day (mid)	0.034 (0.213)	0.009	3.960	***
Quality	<---	Pasture intake of 3–4 kg/day (first)	0.020 (0.125)	0.009	2.310	0.021
Quality	<---	Combination of grass and legume (mid)	0.043 (0.263)	0.009	4.847	***
Quality	<---	Protein concentrate supplementation (first & late)	0.049 (0.252)	0.011	4.606	***
Quality	<---	Goat balancer (late)	0.024 (0.126)	0.011	2.230	0.026
Quality	<---	LnMilkYield	−0.009 (−0.129)	0.004	−2.301	0.021
Quality	<---	Dairy mix with vitamins and minerals (first, mid & late)	0.031 (0.181)	0.010	3.273	***
LnFAT	<---	Pasture intake of 3–4 kg/day (mid)	0.068 (0.230)	0.015	4.410	***
LnFAT	<---	Pasture intake of 3–4 kg/day (first)	0.050 (0.171)	0.015	3.328	***
LnLACT	<---	LnMilkYield	0.020 (0.281)	0.004	5.694	***
Concentrate provided: 300–500 g (mid & late)	<---	Goat balancer (late)	0.202 (0.169)	0.071	2.836	0.005
Dairy mix with vitamins and minerals (first, mid & late)	<---	Pasture intake of 3–4 kg/day (first)	0.113 (0.122)	0.056	2.033	0.042

* Standardized in parenthesis, *** significance at *p* < 0.001.

**Table 7:** SEM results: Regression weights and significance of management covariates.

Affected Variables	Direction of the Effect	Covariates	Estimate	S.E.	C.R.	*p*
Concentrate provided: 300–500 g (mid & late)	<---	Quantity of cereals/concentrates adjusted according to dairy production history	0.303 (0.301) *	0.059	5.110	***
Protein concentrate supplementation (first & later)	<---	Quantity of cereals/concentrates adjusted according to dairy production history	0.246 (0.300)	0.048	5.124	***
Dairy mix with vitamins and minerals (first, mid & late)	<---	Energy supplements used to guarantee adequate energy intake during late pregnancy	0.292 (0.308)	0.057	5.071	***
Protein concentrate supplementation (first & later)	<---	Energy supplements used to guarantee adequate energy intake during late pregnancy	0.205 (0.244)	0.049	4.163	***
Goat balancer (late)	<---	Energy supplements used to guarantee adequate energy intake during late pregnancy	0.227 (0.263)	0.053	4.284	***
Quality	<---	LnAge	0.027 (0.126)	0.012	2.233	0.026
LnFAT	<---	LnAge	0.077 (0.191)	0.020	3.765	***
LnMilkYield	<---	LnAge	1.000 (0.315) *			
Quality	<---	Lactation periods	0.013 (0.133)	0.006	2.358	0.018
Concentrate provided: 300–500 g (mid & late)	<---	Lactation periods	−0.118 (−0.186)	0.038	−3.132	0.002
Protein concentrate supplementation (first & later)	<---	Lactation periods	−0.074 (−0.145)	0.030	−2.508	0.012
Goat balancer (late)	<---	Lactation periods	−0.086 (−0.162)	0.032	−2.682	0.007
Combination of grass and legume (mid)	<---	Lactation periods	0.110 (0.181)	0.039	2.859	0.004
LnLACT	<---	Frequency of mastitis	−0.018 (−0.122)	0.007	−2.480	0.013

* Standardized in parenthesis, *** significance at *p* < 0.001.

**Table 8:** Percentage change in milk components based on unstandardized total effects.

Variables	Protein (%)	Fat (%)	SNF (%)	Lactose (%)
Dairy mix (all lactation)	3.15	2.74	1.51	0.80
Goat balancer (late)	3.56	3.15	1.71	0.90
Protein concentrates	5.02	4.29	2.33	1.21
Grass–legume mix (mid)	4.40	3.77	2.02	1.11
Pasture 3–4 kg (first)	2.33	7.36	1.11	0.60
Pasture 3–4 kg (mid)	3.46	10.19	1.61	0.90
Concentrate 300–500 g (mid & late)	5.97	5.13	2.84	1.51
LnMilkyield *	−0.009	−0.008	−0.004	0.018
LnAge *	0.019	0.093	0.009	0.025

* Continuous log-transformed variables; coefficients are elasticities and are reported without exponential transformation.

## Data Availability

The data supporting the findings of this study are available from the corresponding author upon reasonable request. Data will be provided to enable verification and reproduction of the analyses presented in this article, subject to the protection of participant confidentiality and ongoing related research.
